# New Insight on *In Vitro* Biological Activities of Sulfated Polysaccharides from Ulvophyte Green Algae

**DOI:** 10.3390/molecules28114531

**Published:** 2023-06-02

**Authors:** Fahrul Nurkolis, Rudy Kurniawan, Isma Kurniatanty, Moon Nyeo Park, Myunghan Moon, Siti Fatimah, William Ben Gunawan, Reggie Surya, Nurpudji Astuti Taslim, Hangyul Song, Bonglee Kim

**Affiliations:** 1 Department of Biological Sciences, State Islamic University of Sunan Kalijaga (UIN Sunan Kalijaga), Yogyakarta 55281, Indonesia; fahrul.nurkolis.mail@gmail.com (F.N.); isma.kurniatanty@uin-suka.ac.id (I.K.); siti.fatimah91@uin-suka.ac.id (S.F.); 2 Alumnus of Internal Medicine, Faculty of Medicine, University of Indonesia—Cipto Mangunkusumo Hospital, Jakarta 10430, Indonesia; rudycrates@gmail.com; 3 Department of Pathology, College of Korean Medicine, Kyung Hee University, Seoul 02447, Republic of Korea; mnpark@khu.ac.kr (M.N.P.); audgksdl5364@khu.ac.kr (M.M.); 4 Korean Medicine-Based Drug Repositioning Cancer Research Center, College of Korean Medicine, Kyung Hee University, Seoul 02447, Republic of Korea; 5 Department of Nutrition Science, Faculty of Medicine, Diponegoro University, Semarang 50275, Indonesia; wbwilliambenwb@gmail.com; 6 Department of Food Technology, Faculty of Engineering, Bina Nusantara University, Jakarta 11480, Indonesia; reggie.surya@binus.edu; 7 Division of Clinical Nutrition, Department of Nutrition, Faculty of Medicine, Hasanuddin University, Makassar 90245, Indonesia; pudji_taslim@yahoo.com; 8 Nneul 365 Korean Medical Clinic, Incheon 22397, Republic of Korea; shg3811@khu.ac.kr

**Keywords:** antioxidant activity, sulfated polysaccharides, Indonesian green algae, anti-obesity, antidiabetic, anticancer, Caulerpa, antiproliferative, biological activity

## Abstract

Green algae are natural bioresources that have excellent bioactive potential, partly due to sulfated polysaccharides (SPs) which are still rarely explored for their biological activities. There is currently an urgent need for studies exploring the anticancer biological activity of SPs extracted from two Indonesian ulvophyte green algae: the sulfated polysaccharide of *Caulerpa racemosa* (SPCr) and the sulfated polysaccharide of *Caulerpa lentillifera* (SPCl). The method of isolating SPs and their assessment of biological activities in this study were based on previous and similar studies. The highest yield sulfate/total sugar ratio was presented by SPCr than that of SPCl. Overall, SPCr exhibits a strong antioxidant activity, as indicated by smaller EC_50_ values obtained from a series of antioxidant activity assays compared to the EC_50_ values of Trolox (control). As an anti-obesity and antidiabetic, the overall EC_50_ value of both SPs was close to the EC_50_ of the positive control (orlistat and acarbose). Even more interesting was that SPCl displayed wide-ranging anticancer effects on colorectal, hepatoma, breast cancer cell lines, and leukemia. Finally, this study reveals new insights in that SPs from two Indonesian green algae have the potential to be promising nutraceuticals as novel antioxidative actors, and to be able to fight obesity, diabetes, and even cancer.

## 1. Introduction

One of the most commonly observed green algae in tropical and subtropical marine environments is the ulvophyte algae *Caulerpa*, a genus that includes both *Caulerpa racemosa* and *Caulerpa lentillifera*. In Southeast Asian nations including Japan, Korea, Indonesia, Thailand, and Malaysia, *Caulerpa* has been well-recognized as a wholesome dish [1]. *C. racemosa* has high levels of proteins (8.8–19.9%), essential amino acids (45.28–0.12%), and monounsaturated and polyunsaturated fatty acids, which make up 56.2% of the total fatty acid composition, thereby dominating its fatty acid profile [2]. Another study stated that *C. lentillifera* from Thailand, Malaysia, and China, has a saccharide content of 43.22–59.27%, a total protein content of 12.49–19.38%, and a polyunsaturated fatty acid content of 9.49–29.98%, respectively, as well as safe mineral levels [3]. In addition to being a strong source of vitamins, minerals, and vital trace elements, members of the genus *Caulerpa* are also abundant in pigments such as chlorophyll-a, chlorophyll-b, beta-carotene, and caulerpin [1,4,5,6]. Caulerpa is not only nourishing but is also abundant in significant chemicals that could be used as medicinal agents.

One of the versatile, applicable contents of green algae are sulfated polysaccharides (SPs). SPs are important compounds in the food, pharmaceutical, and cosmetic industries, with benefits supported by an increasing number of studies regarding their application as nutraceutical and pharmaceutical agents [7]. Extracted SPs also exhibit a wide range of properties that are beneficial to health. Extracted SPs of Brazilian *C. racemosa* and cell lines of the SPs of the Chinese *C. lentillifera* have been associated with reduced inflammation in animal models [8,9]. SPs from Brazilian green algae have also exhibited roles in the suppression of fat accumulation, showcasing antiadipogenic activity effects [9], improved enzymatic antibacterial activity [10], neuroprotective potentials [11,12], modulation of immunity [13], antitumor activities [12,14], and hypoglycemic activity [15]. Anti-oxidative stress is important and intricately related to cancer, as with antioxidants it can fight free radicals and also suppress the growth of cancer cells. This was proven by previous studies which demonstrated that SPs from *Sargassum tenerrimum* exhibit antioxidant and anticancer activity [16]. However, studies on SPs from green algae are still limited. Interestingly, environmental factors in the local habitat of each algae affect the conditions of secondary metabolites produced as a means of protection against predation [17]. Each environment has different water conditions, resulting in the production of different secondary metabolites in each algae or seaweed. As *Caulerpa* is a rich repository of bioactive metabolites and high biomass production [5,18], determining the characteristics of these SPs in *C. racemosa* and *C. lentillifera* from various water areas is a topic of interest.

To the best of our knowledge, no study has characterized sulfated polysaccharides (SPs) from the two Indonesian ulvophyte green algae, *Caulerpa racemosa,* and *Caulerpa lentillifera,* and their health implications. To address this information gap, this study aimed to identify the characteristics and health benefits of SPs extracted from Indonesian *C. racemosa* and *C. lentillifera* in terms of antioxidant, anti-obesity, antidiabetic, and anticancer capacities; results were drawn from *in vitro* assays. Furthermore, there have been no studies comparing the anticancer biological activities of the two *Caulerpa* species addressed in the current study, and therefore the benefits of both of them will be known simultaneously. Only a few studies have succeeded in presenting the nature of the SPs of the global green algae *Caulerpa*. As discussed above, the development of research for biological activities also has to be further explored to gain new insights in the field of health and functional food, rather than only focusing on its characterization; this is new information that the researchers have managed to present in this reported work.

## 2. Results

### 2.1. Characterization and Composition of SPs Samples

Sulfated polysaccharides extracted from two Indonesian ulvophyte green algae (*Caulerpa racemosa* and *Caulerpa lentillifera*) were successfully profiled for their yield (%) and sulfate-to-total-sugar ratio, although the protein content percentage was not detected, as summarized in Table 1 (means ± standard deviation). The highest yield was shown by SPCr (28.55 ± 1.00%) followed by SPCl (27.60 ± 2.55%). The SPCr sulfate-to-total-sugar ratio was greater than that of SPCl (0.55 ± 0.01 > 0.40 ± 0.05). SPCr consisted of arabinose, rhamnose, xylose, and mannose, while in SPCl only xylose, rhamnose, and galactose were observed; their structural visualizations are shown in Table S1.

### 2.2. SPCr and SPCl Exhibit Promising Antioxidant Potential

**Figure 1:**
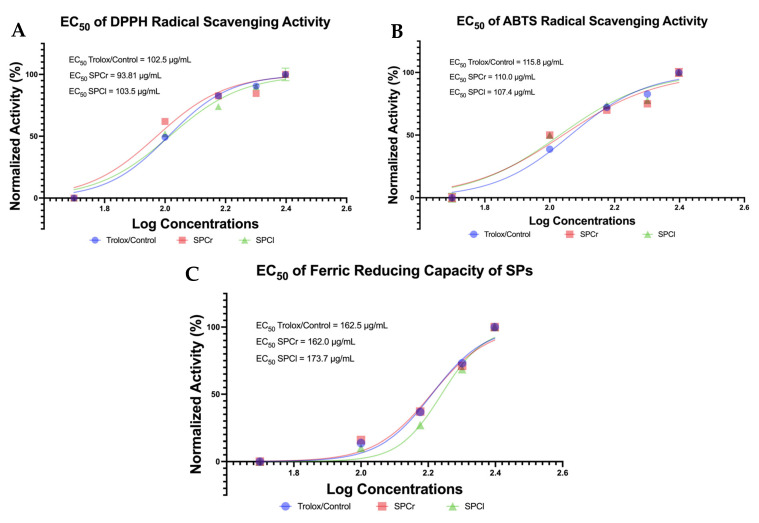
Antioxidant properties of two extracted SPs from Indonesian ulvophyte green algae. (**A**): EC_50_ of DPPH radical scavenging activity. (**B**): EC_50_ of ABTS radical scavenging activity. (**C**): EC_50_ of the ferric reducing capacity of the SPs. SPs: sulfated polysaccharides; SPCr: sulfated polysaccharide of *Caulerpa racemosa*; SPCl: sulfated polysaccharide of *Caulerpa lentillifera*; and Trolox/Control: standard antioxidant used as a positive control.

Free radical scavenging activity and ferric-reducing capacity determine antioxidant properties due to redox properties (oxidation–reduction reactions). Therefore, it is important to observe the potential of SPCr and SPCl extracted from Indonesian green algae specimens as nutraceutical and pharmaceutical candidates in reducing various non-communicable diseases (e.g., obesity, diabetes, and cancer) by improving oxidative stress from reactive oxygen species (ROS). The biological activity of free radical scavenging by SPCr and SPCl was successfully determined using ABTS and DPPH inhibition and FRAP assays. The doses were observed in a dose-dependent manner; the half-maximal effective concentration (EC_50_) is summarized in Figure 1.

Overall, the EC_50_ values of SPCr on DPPH (93.81 μg/mL) and ABTS (110.0 μg/mL) inhibition and FRAP assays (162.0 μg/mL) were lower than for the positive controls (Trolox; 102.5 μg/mL, 115.8 μg/mL, and 162.5 μg/mL, respectively), suggesting agreat potential of SPCr as an antioxidant agent. In addition, SPCl also showed great antioxidant potential in ABTS radical scavenging activity, as the EC_50_ value of SPCl (107.4 μg/mL) was lower than the EC_50_ of Trolox (115.8 μg/mL; positive control). Additionally, the EC_50_ values of SPCl in DPPH inhibition (103.5 μg/mL) and FRAP assays (173.7 μg/mL) were also close to the corresponding EC_50_ of Trolox, which served as a positive control (Figure 1).

### 2.3. Anti-Obesity Potential of Two Extracted SPs from Indonesian Green Algae

**Figure 2:**
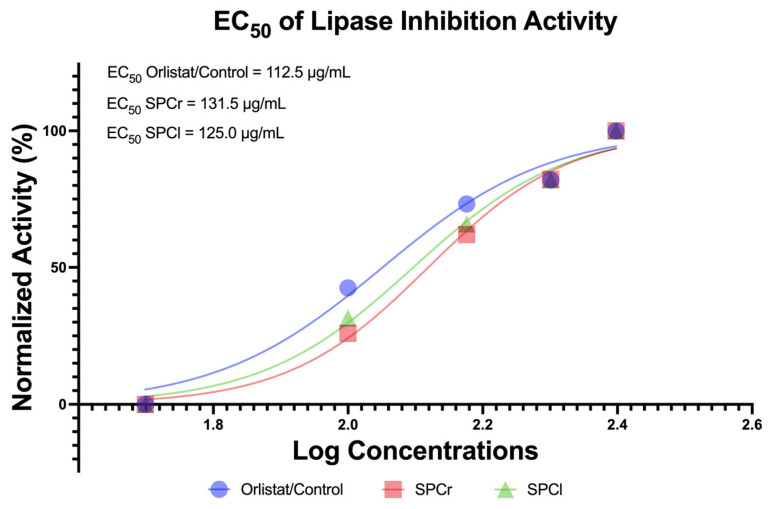
Anti-obesity properties of SPCr and SPCl via lipase inhibition. SPCr: sulfated polysaccharide of *Caulerpa racemosa*; SPCl: sulfated polysaccharide of *Caulerpa lentillifera*; and Orlistat/Control: standard drug serving as a positive control.

*In vitro* anti-obesity effects were mainly determined by observing the inhibitory properties of the samples against a lipase enzyme (lipid hydrolyzing enzyme). As nutraceutical and pharmaceutical candidates, the anti-obesity properties of SPCr and SPCl were determined through the inhibition of lipase. The lipase inhibition concentrations were observed in a dose-dependent manner, and the half-maximal effective concentration (EC_50_) was highlighted as shown in Figure 2.

According to the EC_50_ data presented above in Figure 2, in general, the EC_50_ values of the two SPs were close to the EC_50_ level of the standard drug orlistat, the positive control: EC_50_ orlistat (112.5 μg/mL) < SPCl (125.0 μg/mL) < SPCr (131.5 μg/mL). Interestingly, the results of the ANOVA test revealed that there was no significant differences between the lipase inhibition activity of the SPs and the controls (*p* > 0.05; Table S2).

### 2.4. The Promising Antidiabetic of Two Isolated SPs from Indonesian Green Algae

**Figure 3:**
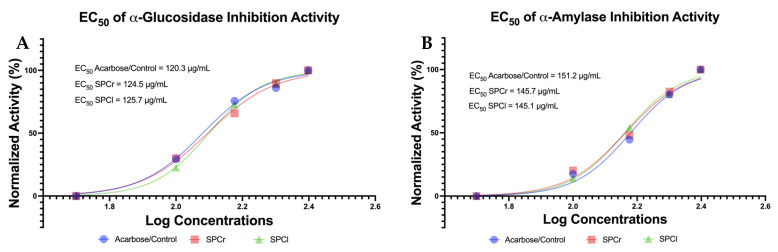
Antidiabetic properties of SPCr and SPCl. (**A**) EC_50_ of α-glucosidase inhibition activity. (**B**) EC_50_ of α-amylase inhibition activity. SPCr: sulfated polysaccharide of *Caulerpa racemosa*; SPCl: sulfated polysaccharide of *Caulerpa lentillifera*; and Acarbose/Control: standard drug used as a positive control.

The inhibitory activity of α-glucosidase and α-amylase determines the antidiabetic properties of both SPs, as this is based on their inhibitory properties against the carbohydrate hydrolyzing enzymes themselves (α-glucosidase and α-amylase). This is important for observing the potential of SPCr and SPCl as functional food candidates in reducing metabolic syndrome diseases, especially diabetes. Antidiabetic potential in this study was evaluated based on *in vitro* α-amylase and α-glucosidase inhibition activity in a dose-dependent manner (Figure 3).

The EC_50_ value of α-amylase inhibition activity markedly revealed the promising potential of the two SPs extracted from Indonesian green algae compared to a positive control (acarbose). EC_50_ values of α-amylase inhibition activity were sorted from lowest to highest as follows: SPCl (145.1 μg/mL) < SPCr (145.7 μg/mL) < acarbose (151.2 μg/mL). However, the EC_50_ values of α-glucosidase inhibition activity were 124.5 μg/mL (SPCr) and 125.7 μg/mL (SPCl), respectively, which is close to the acarbose’s EC_50_ value of 120.3 μg/mL. Interestingly, the results of the ANOVA test showed that there was no significant difference between the α-glucosidase and α-amylase inhibition activities of these SPs and the controls (*p* > 0.05; Table S2).

### 2.5. Promising Anticancer Properties of Two Extracted SPs from Indonesian Green Algae

Apart from being antioxidant, anti-obesity, and antidiabetic agents, SPCr and SPCl also hold the potential for anticancer activity in several cancer cells, as summarized in Table 2. For example, SPCr had IC_50_ values that imply a strong activity against hepatoma cancer cells (Hep G2 [175.10 μg/mL] and KAIMR C1 [320.10 μg/mL]) and breast cancer cell lines (MDA-MB-231 [550.60 μg/mL]; MCF-7 [201.50 μg/mL]; and KG-1a [403.90 μg/mL]). Notably, SPCl demonstrated a wide range of anticancer activity, particularly against colorectal anticancer (IC_50_: 482.10 μg/mL), hepatoma (IC_50_: 401.40 μg/mL in KAIMR C1), breast cancer cell lines (IC_50_: 310.90 μg/mL in MDA-MB-231; and 330.53 μg/mL in MCF-7, respectively), and leukemia (IC_50_: 306.90 μg/mL). Cytotoxicity to normal monocular blood (PBMC) and human epithelial cell lines was observed, and the two extracted SPs from Indonesian green algae (SPCr and SPCl) were categorized as safe functional food candidates (IC_50_ > 1000 μg/mL). This suggests that not only do SPCr and SPCl have promising biological activity, but they are also safe against normal cells and safe to consume.

### 2.6. Anti-Ageing Capabilities of SPs via Modulation of mTOR-SIRT1-AMPK Pathways

**Figure 4:**
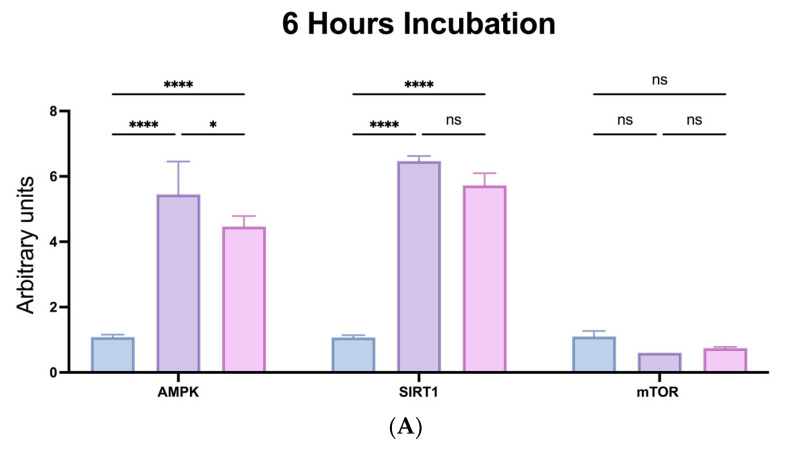

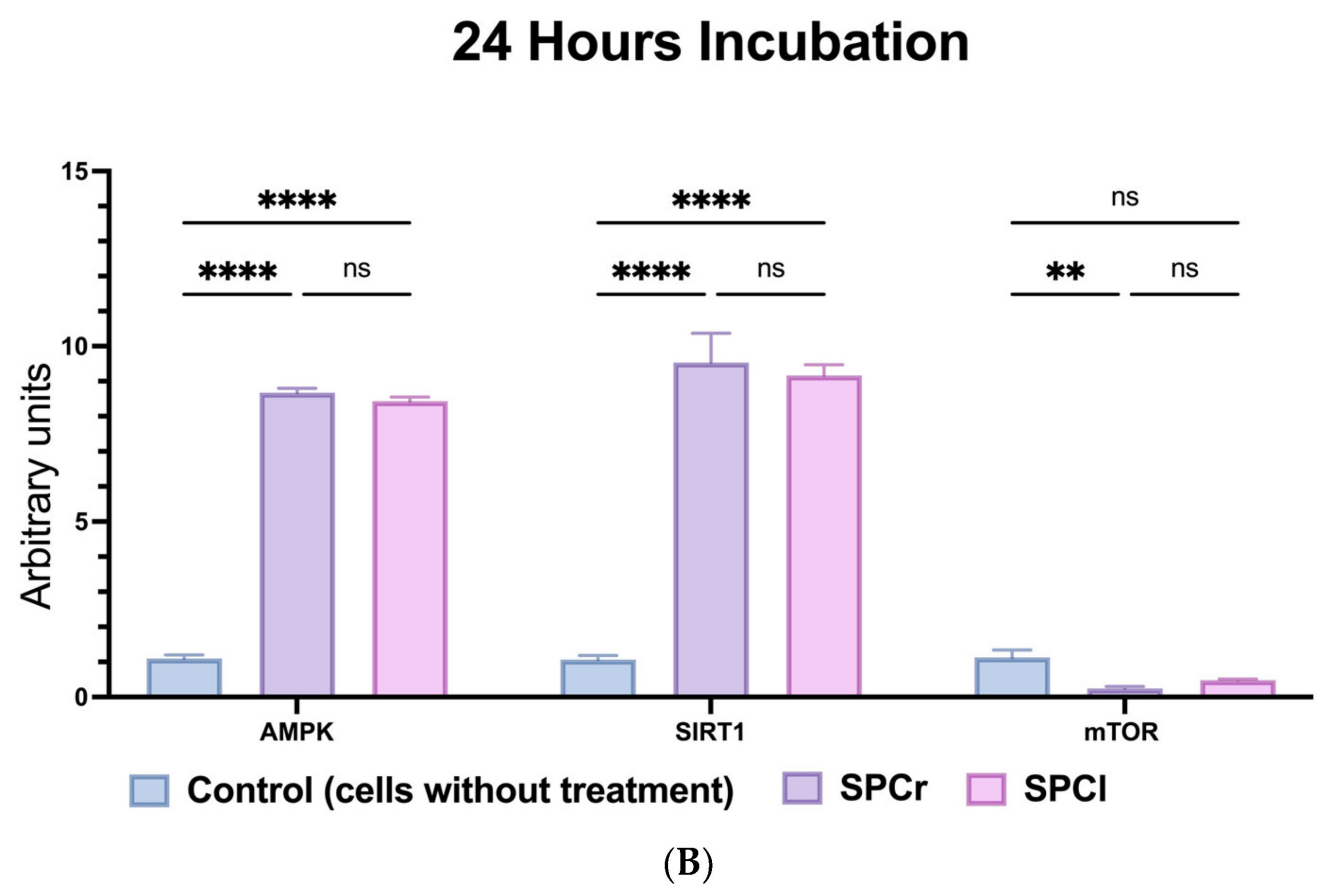
The expression of the mammalian target of rapamycin (mTOR) kinase, AMP-activated protein kinase (AMPK), and sirtuin 1 (SIRT1) regulated by SPCr and SPCl. (**A**) Expression at 6 h of incubation. (**B**) Expression at 24 h of incubation. **** *p* < 0.0001, ** *p* = 0.0079, * *p* = 0.0149, and ^ns^ *p* > 0.05.

At 6 and 24 h of incubation, the expression of AMPK and SIRT1 was found to be significantly elevated in both SPs compared to the control (*p* < 0.0001) (Figure 4). The sulfated polysaccharide of *Caulerpa racemosa* (SPCr) demonstrated a stronger effect on the increase of AMPK expression compared to the sulfated polysaccharide of *Caulerpa lentillifera* (SPCl) (*p* = 0.0006) in 6 h of incubation (Figure 4A). In line with the AMPK result, SPCr showed a greater impact on the expression of SIRT1 compared to SPCl, but no significant difference was found (*p* > 0.05). On the other hand, both SPCr and SPCl did not cause significant changes in mTOR expression at six hours, except in 24 h of incubation whereby SPCr significantly decreased mTOR expression compared to the control (*p* = 0.0079) (Figure 4B).

## 3. Discussion

Marine algae contain molecular bioresources or metabolites with many biological activities that have proven to be interesting and may have the potential to be developed into nutraceutical agents and pharmaceuticals [19,20]. Unfortunately, the vast and diverse group of marine algae remains minimally explored, including regarding the evidence of its biological activity. More specifically, the biological activity of SPs extracted from Indonesian green algae has not been previously described, even though it has the potential for biomedical agent applications. Marine algae present more complex and diverse chemicals and metabolites than other algae or seaweeds, and they are widespread in the environment [21]. Considering the biological properties and availability of green algae, they therefore represent an interesting natural resource for further study and development as nutraceutical agents and pharmaceuticals. In this context, this study successfully reported their characteristics, and explored the health benefits of SPs extracted from Indonesian *C. racemosa* and *C. lentillifera* in terms of their antioxidant, anti-obesity, antidiabetic, and anticancer properties. In addition, the biological activities of two SPs extracted from Indonesian green algae (SPCr and SPCl) have been successfully compared. The composition of monosaccharides in brown algae or brown seaweed (*Sargassum tenerrimum*) by Raguraman *et al.* (2019) compared to current research (two Indonesia green algae) is largely the same, which all comprise galactose, mannose, and arabinose [16]. However, the fundamental difference is that in the current study (evaluating SPCr and SPCl), xylose monosaccharide was observed while fucose monosaccharide (which is widely observed in most brown seaweed) was not [16,22]. Furthermore, in the current study (SPCr and SPCl), type B ulvanobiuronic acid 3-sulfate and type A ulvanobiuronic acid 3-sulfate were observed, which have never been reported to be found in brown algae. Polysaccharides in green algae come from their own starch-cell walls, while sulfated ones are thought to arise from natural sulfation [8,10,13,14]. Essentially, sulfate is present in the amino acids cysteine and methionine and in a large number of essential coenzymes and cofactors [23]; it is also a constituent of sulfate esters in many cellular metabolites, carbohydrates [23], and proteins present in algae. However, relatively little is known regarding the function of most sulfated metabolites, and the synthesis of activated sulfates used in the sulfation pathway is especially important for future studies.

In terms of anti-oxidative properties, the two SPs from extracted Indonesian green algae (SPCr and SPCl) both exhibit promising antioxidant activity, as their EC_50_ values were found to be more potent than Trolox, which served as a positive control (Figure 1). Even when compared to studies of the antioxidant activity of SPs from African *Gracilaria gracilis* and *Ulva lactuca* [11], the EC_50_ values of SPCr and SPCl were more potent. This study showed that the highest EC_50_ of antioxidant activity was 333.33 μg/mL. Furthermore, another study found that the sulfated polysaccharide of *Sargassum tenerrimum* was lower by more than 50% compared to the highest concentration (125 μg/mL) [16]. However, all work, including the current study, was conducted in a dose-dependent manner. Evidence-based reports from meta-analyzes suggest that antioxidant treatment is clinically effective for diabetes mellitus and associated complications of obesity [24], and potentially also inhibits the growth of cancer cells [25]. In line with this, the present study also determined anti-obesity activity based on the inhibition of lipid-hydrolyzing enzymes (lipase inhibition). Obesity has become an epidemic globally, and its prevalence has been predicted to continue to rise, with notably higher rates estimated by 2030 [26]. Obesity is characterized by an abnormal lipid accumulation attributed to lipase activity [27], but the physiological consequences of obesity are much more concerning and can require treatment that is still expensive [28,29]. Marine bioresources, including SPCr and SPCl, may be promising nutraceutical and pharmaceutical anti-obesity agents. The anti-obesity results obtained from this study are in line with the findings reported by Chaves Filho et al., which indicated that the SPs of Brazilian green algae also showed suppression of fat accumulation, thereby suggesting an antiadipogenic effect [9]. Until now, no studies have successfully reported on the anti-obesity activity of the SPs of green algae via lipase inhibition. In this current study, it has succeeded in presenting new evidence of the anti-obesity activity of the SPs extracted from green algae (SPCr and SPCl) via lipase inhibition, and based from their EC_50_ values, have the potential to become agents of anti-obesity and related diseases (Figure 2).

Furthermore, there is reason to believe that there is a relationship between obesity with insulin resistance and pancreas β-cell dysfunction in diabetics [30,31]. Therefore, there is a need to study new approaches to managing and preventing diabetes in obese individuals based on natural ingredients that are more sustainable and inexpensive, and these should be investigated based on evidence and facts [32]. Reinforced by evidence from other studies, SPs extracted from green algae have been shown to effectively inhibit the aggregation of human islet amyloid polypeptide and demonstrate a strong antidiabetic activity [15], although this was of a different genus than *Caulerpa*. Until now, there have been no studies that have successfully reported on the antidiabetic activity of green algae SPs via their inhibition of carbohydrate hydrolyzing enzymes (α-glucosidase and α-amylase), something that has been accomplished in this study. Inhibition of α-glucosidase and α-amylase has become an evidence-based antidiabetic determination method, as the inhibition of α-glucosidase and α-amylase can delay glucose absorption and lower post-prandial blood glucose levels, thus making this process a management strategy for type 2 diabetes [33,34]. Interestingly, SPs extracted from Indonesian green algae (SPCr and SPCl) managed to exhibit their antidiabetic activity through α-glucosidase and α-amylase inhibition (Figure 3). This study succeeded in presenting a new inhibitor of α-glucosidase and α-amylase with an EC_50_ value that is more potent than the controls or the standard drugs for diabetes (acarbose; Figure 3), and this is in line with its antioxidant and anti-obesity activities. Oxidative stress is important and is related to both cancer and obesity, and the findings of this research (SPCr and SPCl) are in line with the study of Wang and Cheong (2023), which reported that marine polysaccharides display a good activity in fighting free radicals, suppressing the growth of cancer cells [16], and also improve hyperlipidemia [35]; all of which are in line with the lipase inhibition activity of SPCr and SPCl.

One of the global problems posing a danger to human health today is cancer, which has a direct, significant correlation with mortality. Global cancer data shows that there were 10.0 million cancer deaths (9.9 million excluding nonmelanoma skin cancer) and 19.3 million new cases globally in 2020 [36,37]. As a result, the development of drugs targeting specific cancer-related targets, in conjunction with a thorough understanding of how drugs interact with human tumor biology, has emerged as a key to the current search for a cure for cancer. Despite the huge efforts of current research, effective cancer treatments are still lacking, indicating a need for other sources of cancer treatment agents, such as natural sources. Natural sources, especially underutilized seafood, have a promising potential as functional or nutraceutical foods with anticancer properties. In the present study, the extensive and promising anticancer activity of SPs extracted from two ulvophyte green algae, *C. racemosa* and *C. lentillifera*, has been successfully observed. As shown in Table 2, SPCr and SPCl displayed good inhibitory activity against colorectal, hepatoma, breast cancer, and leukemia cell lines, something which has not been reported in similar studies. This was reinforced by studies on the green algae species *Codium bernabei* [12], which exhibits anticancer activities on HCT-116, MCF-7, and HL-60 cells, but again, this is of a different genus than *Caulerpa*. Of note, apart from being promising anticancer agents, SPCr and SPCl also exhibit less cytotoxic properties in normal cells and are considered safe, since the lethal concentration (IC_50_) is >500 μg/mL (Table 2), based on the National Cancer Institute criteria [20].

Based on the data reported in this study, it was highlighted that the synergistic effects of SIRT1 and AMPK may contribute to the greater anti-aging activities of *C. racemosa* and *C. lentillifera* SPs, and also by modulatory of mTOR expression. Our results provide evidence that both SPs have the potential ability to stimulate SIRT1, which has not been widely reported in the literature, and this is a new insight into this topic area. In this regard, we also confirmed the interaction of these SPs with the AMPK and mTOR pathways, which is in support of a study that highlights the importance of the SIRT1/AMPK and mTOR pathways in many diseases, particularly aging-related diseases [38]. This is in line with and supports previous research examining the role of nutraceutical SIRT1 modulators (natural products) in the AMPK and mTOR pathways through evidence of their synergistic effects [39]. SPCr and SPCl have promising antioxidant, anti-obesity, antidiabetic, anti-aging, and anticancer activities, and can be developed as new drugs and functional foods supported by cytotoxicity that are safe against normal cells, as shown in Table 2.

Sulfated polysaccharides (SPs) from two Indonesian ulvophyte green algae (SPCr and SPCl) can be a new alternative source of sulfated polysaccharides with several activities against non-communicable diseases, such as obesity, cancer, diabetes, and aging (based on *in vitro* studies), which can also meet the needs of sulfated polysaccharides apart from brown algae. Due to the limited study of the health benefits of SPs extracted from the ulvophyte green algae of *Caulerpa*, especially from Indonesian seawater, there are also limited comparative discussions presented in this study. Nevertheless, this study is of novel value, and complements the health property profiles of SPs extracted from two ulvophyte green algae in the genus *Caulerpa*. As the current study was conducted *in vitro*, follow-up *in vivo* studies, as well as human clinical trials, are essential to strengthen the evidence of the health benefits of these SPs and have been planned by the authors. In addition, in terms of implications for the future, it will be necessary to analyze the antibacterial and antiviral potential of SPCr and SPCl so that their health benefits can be comprehensively known. In future studies, it is especially important to conduct a purification process where each detected polysaccharide is expected to reach >95% (NMR and FTIR follow-up analysis) before performing animal trials, which is actually a limitation of our current study. This purification process and animal testing have been planned to be conducted in conjunction with animal trials or preclinical trial studies. Furthermore, research to develop SPs into prebiotics, as well as synbiotic products paired with their biological activities including gut microbiome regulation, is an interesting point of assessment to be conducted in the future.

## 4. Materials and Methods

This experimental research was conducted jointly between UIN Sunan Kalijaga (F.N.) and Hasanuddin University (N.A.T.) and supported by the Korean Medicine-based Drug Repositioning Cancer Research Center, College of Korean Medicine, Kyung Hee University, Republic of Korea (B.K. and M.N.P.).

### 4.1. Preparation of the Indonesian Green Algae Samples

Fresh samples of both green algae (*Caulerpa racemosa* and *Caulerpa lentillifera*; or sea grapes, in other terms) were collected from Indonesian sea waters (1° 37’ 6.9924” N, 124° 45’ 59.616” E; North Sulawesi or North Sulawesi, Indonesia), which was approved by local authorities. Botanical identification and authentication were conducted based on previous study protocols [20], and in compliance with the National Center for Biotechnology Information (NCBI) Taxonomy ID database (NCBI:txid148947 and NCBI:txid76317), and the Integrated Taxonomic Information System (Report ID 6968 and 6973). All relevant *in vitro* and algae research guidelines were referred to by the authors (researchers) while conducting this work. Whole green algae were cleaned and then dried at 50 °C using the IN55 Memmert incubator oven (Schwabach, Germany) and adhering to the previous study protocol. The two dried simplicia (*C. racemosa* and *C. lentillifera*) were then ground using a blender to reduce their size and macerated for 24 h with acetone solvent, resulting in a macerated powder (Graphical Abstract).

### 4.2. Isolation and Characterization of Sulfated Polysaccharides from Indonesian Caulerpa

The SP isolation protocol was conducted based on a previous, similar study [40] via enzymatic extraction by the protease enzyme. For 16 h (under agitation) at a temperature of 60 °C and pH 8.0, as much as 50 g of each macerated algae powder was suspended in two volumes of 200 mL sodium chloride (NaCl; 0.25 M) and incubated with Alcalase^®^ (protease from *Bacillus licheniformis*, Subtilisin A; Merck, Darmstadt, Germany). Under gentle agitation, the combined mixture was then filtered using a thin filter cloth and precipitated with 2.0 volumes of ice-cold MeOH (methanol) and the resulting products were stored in a refrigerator at 4 °C for 12 h. The crude extract (precipitate) was collected, centrifugated for 20 min (10,000× *g*), and vacuum-dried, resulting in the SP. The SP of *Caulerpa racemosa* (SPCr) and the SP of *Caulerpa lentillifera* (SPCl) then underwent characterization and determination of their biological activity (Graphical Abstract).

Characterization of total methylated-sugar concentrations was estimated with a phenol–H_2_SO_4_ reaction using volumetric-chromatography procedures, as proposed by Chaves Filho [40], using d-galactose (C_6_H_12_O_6_; PubChem CID: 6036) as the standard. After the polysaccharide underwent acid hydrolysis (6 N HCl, 100 °C, 4 h), the sulfate and monosaccharide content was measured with the Barium chloride-gelatin method spectrophotometrically (turbidimetric measurements using a high-performance liquid chromatography (equipped with a VisionHT C18 column: 250 mm × 4.6 mm, 5 µm) and a UV-visible spectrophotometer detection combined with mass spectrometry (HPLC-UV-Vis/MS; 274 nm detector), and the protein content as described previously [40,41]. Prior to the HPLC test, approximately 10 mg of the crude sulfated polysaccharide extracts (SPCr and SPCl) were hydrolyzed and predigested with trifluoroacetic acid (TFA, 2 mL, 2 M) at 120 °C for 60 min. After hydrolysis, the reaction medium was dried with a vacuum concentrator using distilled water as a solvent. Then, the resulting solution was neutralized using NaOH (1N; pH 7.0). The monosaccharide standards L-Rhamnose (L-Rha), D-Mannose (D-Man), D-Galactose (D-Gal), D-Xylose (D-Xyl), and D-Arabinose were all determined using the same method.

### 4.3. Antioxidant Activity by DPPH and ABTS Radical Scavenging Activity, and FRAP (Ferric Reducing Antioxidant Power) Assay

Antioxidant activity in the 2,2-diphenyl-1-picrylhydrazyl radical scavenging activity ([DPPH radical, C_18_H_12_N_5_O_6_^+^], BioVision, Milpitas, CA, USA) test was assayed according to the protocol of Permatasari [42]. In the testing vial, concentrations of 50, 100, 150, 200, and 250 μg/mL of the SPCr and SPCl samples were added to the DPPH reagent (3 mL). The DPPH-sample mixtures were then cooled at room temperature for 30 min. Changes in the concentration of DPPH were observed based on a 517 nm absorbance.

The scavenging of 2,2′-Azino-bis(3-ethylbenzothiazoline-6-sulfonic acid) ([ABTS+, C_18_H_24_N_6_O_6_S_4_] Sigma-Aldrich, Darmstadt, Germany) or diammonium salt radical cation were determined based on the procedure by Permatasari [42]. Potassium persulfate (K_2_S_2_O_8_ 2.4 mM) and 7 mM ABTS were mixed at a ratio of 1:1, protected from light with aluminum foil, and allowed to react at 22 °C for 14 h. The mixture was further diluted (e.g., 1 mL of the stock solution plus 60 mL of EtOH [ethanol, C_2_H_6_O]) to obtain a working solution with an absorbance of 0.706 at 734 nm. A fresh working solution was prepared for each test. The (SPCr and SPCl) samples were kept in gradients of 50 μg/mL, 100 μg/mL, 150 μg/mL, 200 μg/mL, and 250 μg/mL, respectively, to be diluted with ABTS working solution (1 mL), and the absorbance was measured after 7 min at 734 nm. Inhibition of DPPH and ABTS was expressed as a percentage (%), and determined according to the formula below:

(1) $$\mathrm{Inhibition}\;\mathrm{Activity}\;\left(\%\right)=\left[\frac{A0-A1}{A0}\right]\times 100\%\;$$

where A0 is absorbance of the blank, and A1 represents the absorbance of the standard or sample.

The FRAP (ferric reducing antioxidant power) test was conducted in accordance with the method established in the literature [43,44]. As a FRAP reagent, 300 mM of sodium acetate buffer (C_2_H_3_NaO_2_, pH 3.6, 10 mL) was added to a 10 mM TPTZ (2,4,6-Tris(4-Pyridyl)Triazine, C_18_H_14_N_6_) solution in 40 mM of hydrochloric acid (ClH, 1 mL) and 20 mM of ferrous (III) chloride (1 mL). FRAP reagents were placed in water baths at a temperature of 37 °C. SPCr and SPCl samples, in a gradient of 50, 100, 150, 200, and 250 μg/mL, respectively, were mixed with FRAP reagents (1 mL). Absorbance was determined at 593 nm immediately. The FRAP value was recorded with the following equation, in which Ac is the positive control absorbance once reacted with the Trolox and FRAP reagents, as does the absorbance of the sample, and Ab is the absorbance of blanks reacted with distilled water and the FRAP reagent.

(2) $$\mathrm{FRAP}\;\mathrm{Value}\;\left(\%\right)=\left[\frac{\mathrm{Ac}-\mathrm{Ab}}{\mathrm{Ac}\;-\mathrm{Ab}}\right]x\;2\;\;\;\;\;\;\;\;\;\;\;\;\;\;$$

To ensure the validity of the resulting data (ABTS, DPPH, and FRAP assays), each sample was performed three times (triplicate tests; *n* = 3). Trolox (C_14_H_18_O_4_; PubChem CID: 40634), a known antioxidant molecule, was used as the positive control in the ABTS, DPPH, and FRAP assays. The half-maximal effective concentration ratio (EC_50_) was used to express the radical scavenging capability of the SPCr and SPCl samples and Trolox, and is defined as the concentration of a sample that caused a 50% decrease in the initial radical concentration.

Inhibition of DPPH and ABTS, as well as FRAP assays was expressed as a percentage and determined according to the formula described in the literature [42,43].

### 4.4. In Vitro Anti-Obesity of SPs via Lipase Inhibition Assay (%)

Crude pig pancreatic lipase (PPL, 1 mg/mL) was first dissolved in 50 mM phosphate buffer (pH 7) before being centrifuged at 12,000× *g* to remove insoluble components. The creation of an enzyme stock (0.1 mg/mL) required a 10-fold dilution of the supernatant with buffer. The lipase inhibition potential was assessed based on prior research [45]. A transparent 96-well microplate containing 100 µL of SPCr and SPCl samples was combined with 20 µL of 10 mM p-nitrophenyl butyrate (pNPB) in buffer and incubated for 10 min at thirty-seven degrees Celsius (37 °C). The outcome was compared to the reference drug Orlistat (C_29_H_53_NO_5_, PubChem CID: 3034010), a well-known PPL or lipase inhibitor. Measurements were taken at 405 nm using a DR-200Bc ELISA microplate reader (Thermo Fisher Scientific, Waltham, MA, USA). The unit of activity was calculated using the yield of the reaction rate of 1 mol of p-nitrophenol (4-nitrophenol, C_6_H_5_NO_3_) per minute at thirty-seven degrees Celsius (37 °C). To measure the lipase inhibition activity, PPL activity was reduced in the test mixture by a specific amount. To ensure the validity of the study results, each sample was verified in thrice or triplicate (*n* = 3). The inhibitory data were obtained using the equation described previously by Permatasari et al. [45]:

(3) $$\;\;\;\;\;\;\;\;\;\;\mathrm{Inhibition}\;\mathrm{of}\;\mathrm{lipase}\;\mathrm{activity}\;\left(\%\right)=100-\left[\frac{B-\mathrm{Bc}}{A-\mathrm{Ac}}\right]\times 100\%\;\;\;\;\;\;$$

where A represents the activity without inhibitor; B indicates the activity with inhibitor; Ac represents the negative control (-) without inhibitor; and Bc designates the negative control (-) with inhibitor.

### 4.5. In Vitro Antidiabetic Assay via α-Glucosidase and α-Amylase Inhibition Assay (%)

The inhibitory activity was performed according to previous literature [42]. The enzyme (76 UI, 1 mg) was mixed with phosphate buffer (50 mL with pH 6.9) to obtain a concentration of 1.52 UI/mL. In the reaction tube, 0.35 mL of sucrose (65 mM), maltose solution (65 mM), and the samples (SPCr and SPCl; quantities of 0.1 mL, 50 μg/mL, 100 μg/mL, 150 μg/mL, 200 μg/mL, and 250 μg/mL, respectively) were added one at a time. After homogenization, α-glucosidase solution (1.52 UI/mL, 0.2 mL) was added to each tube, which was then maintained at thirty-seven degrees Celsius (37 °C) for 20 min. The enzyme was then inactivated and heated in a water bath for 2 min at 100 °C. Acarbose served as the positive control. To develop the color, 0.2 mL of testing solution and color reagent (3 mL) was added consecutively. Next, the system was warmed up to thirty-seven degrees Celsius (37 °C) for 5 min, and the solution absorption was examined at 505 nm afterward. The amount of glucose released during the reaction served as a marker of inhibitory activity.

The α-amylase (alpha-amylase) inhibition activity of the SPCr and SPCl samples was measured based on previous literature [45,46]. Diluted samples were incubated at five different concentrations (50 μg/mL, 100 μg/mL, 150 μg/mL, 200 μg/mL, and 250 μg/mL, respectively) for 10 min at 25 °C with sodium phosphate buffer (0.02 M, pH 6.9) and 0.006 M NaCl, as well as 0.5 mg/mL porcine pancreatic amylase. Then, 500 μL of a 1% starch solution in assay buffer was added to each mixture. After 10 min of incubation at 25 °C, 3,5-dinitro salicylic acid was added to complete the process and, incubating in a water bath at one hundred degrees Celsius (100 °C) for 5 min, the test tube was allowed to cool to 22 °C. To achieve values in the permissible range for recording the absorbance at 540 nm, dilution with distilled water (10 mL) was performed. The positive control used was acarbose.

### 4.6. Anticancer Evaluation of SPs via Antiproliferative Activity

The protocol study [20,47], in which antiproliferative experiments were conducted was authorized by the author’s institutional review board (IRB). American Type Culture Collection (ATCC), Manassas, VA, USA, provided colorectal cancer (HCT-8), leukemia (acute myeloid leukemia: HL-60; lymphoblastic: K-562; and erythroleukemia: KG-1a), human breast cancer (MCF-7), breast epithelial cancer (MDA-MB-231), hepatic cancer (Hep G2), and normal breast epithelial cell lines (MCF-10A). In the authors’ laboratory, primary blood monocular cells (PBMC) and the KAIMRC 1 cell line were created. The positive control was mitoxantrone (C_22_H_28_N_4_O_6_; CID 4212), which was bought from Sigma-Aldrich^®^.

The CellTiter-Glo^®^ assay (Promega, Madison, WI, USA) was employed in line with the manufacturer’s instructions to ascertain the impact of the samples (SPCr and SPCl) on the proliferation of the non-adherent cells. A Perkin Elmer’s Envision plate reader was used to quantify the luminescence, which was then normalized to average dimethyl sulfoxide (DMSO) controls and represented as a percentage. Each portion of the samples ranged in concentration from 50 μg/mL, 100 μg/mL, 150 μg/mL, 200 μg/mL, and 250 μg/mL, respectively, and the cells were planted in 96-well plates using a growth medium. CellTiter-Glo^®^ reagent was added to each well and stirred for 2 min, and luminescence was measured using an Envision plate reader after the cells were incubated at thirty-seven degrees Celsius (37 °C) for 24 h (PerkinElmer, Inc., Waltham, MA, USA). The half maximal inhibitory concentration or IC_50_ values (g/mL), were computed for each sample [48].

The MTT test was conducted using the aforementioned method. To assess the impact of the SPCr and SPCl samples on the proliferation of adherent cells, the MTT reagent (Sigma-Aldrich^®^, Germany) was used in place of the CellTiter-Glo^®^ reagent. Absorbance was measured using the SpectraMax spectrophotometer from Invitrogen (Waltham, MA, USA). It was then adjusted to average DMSO controls and given as a percentage [49].

### 4.7. In Vitro Assays of Mammalian Target of Rapamycin (mTOR) Kinase, AMP-Activated Protein Kinase (AMPK), and Sirtuin 1 (SIRT1) Expressions

*In vitro* evaluations for mTOR, AMPK, and SIRT1 were referred to the protocol of each kit provider manufacturer, and were in line with the established study protocol [39]. A total of 25 μg/μL of cell lysates were combined with the right amount of 1 SDS sample buffer × (0.5 M Tris-HCl in pH 6.8, 20% SDS, 10% [*v*/*v*] glycerol (C3H_8_O_3_), 5% [*v/v*] β-mercaptoethanol (HOCH_2_CH_2_SH), and 0.2% bromophenol blue), heated at 95 °C for 5 min, separated by SDS-PAGE, and then transferred to a polyvinylidene difluoride membrane. The membrane was blocked with 5% lean dry milk (*w*/*v*) in saline buffered Tris with Tween buffer (T-TBS) (20 mmol/L Tris-HCl, 0.138 mol/L sodium chloride (NaCl) (pH 7.6), and 0.1% Tween 20) for SIRT1, total AMPK, and mTOR detection, with 5% (*w*/*v*) albumin (bovine serum albumin or BSA) in T-TBS for phospo-AMPKα and phospo-mTOR. The expression of SIRT1 and mTOR/AMPK activation was analyzed by incubating the membrane in the presence of specific primary antibodies, and subsequently, secondary peroxidase-conjugated antibodies, suitably diluted in 5% BSA in T-TBS. Primary antibodies were used overnight at 4 °C under the following conditions: Anti-SIRT1, rabbit (1:1000); total-AMPK, rabbit (1:200); antiphospo-mTOR, rabbit (1:1000); antiphospo-AMPKα, rabbit (1:200); and total-mTOR, rabbit (1:1000). Secondary antibodies were used at two different dilutions: goat anti-rabbit (1:10,000) for SIRT1 and total and phospo-mTOR, and goat anti-rabbit (1:2000) for total and phospo-AMPK. More precisely, 5000 cells per well were seeded in the volume of the medium so that the final volume was 100 μL/well. The cells were treated with two SPs extracts (25 μM samples: SPCr and SPCl) and incubated for 6 and 24 h, respectively, and through analysis, obtained percentage values compared to the controls (cells without treatment).

### 4.8. Management and Analysis of Data

All statistical analysis was conducted using GraphPad Prism 9 Premium Software MacBook version; data are shown as calculated means and standard deviation (means ± SD). The EC_50_ was analyzed using the GraphPad Premium statistical analysis package “non-linear regression (log(inhibitor) vs. normalized response-variable slope”, to statistically assess the data from obtained from the *in vitro* experiments, including antioxidant inhibition of DPPH, ABTS, FRAP, lipase, α-glucosidase, and α-amylase, and antiproliferation (anticancer activity), and was performed three times.

## 5. Conclusions

In this study, new insights were reported in that sulfated polysaccharides extracted from two Indonesian ulvophyte green algae (*Caulerpa racemosa* and *Caulerpa lentillifera*) were successfully characterized based on their composition and new insights were also obtained regarding their biological activity profiles. The sulfated polysaccharide of *Caulerpa racemosa* (SPCr) and sulfated polysaccharide of *Caulerpa lentillifera* (SPCl) have the potential to be novel candidates for nutraceuticals and pharmaceutical agents against free radicals and oxidative stress, fat accumulation and obesity-related diseases, and diabetes, and have great promise as anticancer and anti-aging agents in the future. However, it is of utmost importance to conduct follow-up *in vivo* tests and human clinical trials based on the results reported from this study.

## 6. Patents

The SP preparation method with their formulation resulting from the work reported in this article has been registered as a patent with the number S00202301037 in Indonesia.

## Figures and Tables

**Table 1:** Characterization and composition of the SP samples from two Indonesian ulvophyte green algae (*Caulerpa racemosa* and *Caulerpa lentillifera*).

Sample	Yield (%)	Protein (%)	Sulfate-to-Total-Sugar Ratio	* Monosaccharides	Observed Methylated Sugars (Peak Area; MW)
SPCr	28.55 ± 1.00 ^a^	nd	0.55 ± 0.01 ^a^	Arabinose, mannose, rhamnose, and xylose	2,3-di-*O*-methyl-1,4,5-tri-*O*-acetyl arabinitol (20%; 306.31), 2,3,4,6-tetra-*O*-methyl-*D*-mannopyranose (24%; 236.26), and type B ulvanobiuronic acid 3-sulfate (36%)
SPCl	27.60 ± 2.55 ^a^	nd	0.40 ± 0.05 ^a^	Xylose, galactose, and rhamnose	2,3,4-tri-*O*-methyl-1,5-di-*O*-acetyl xylitol (17%; 278.30), 2,3,4,6-tetra-*O*-methyl-*D*-galactopyranose (20%; 236.26), and type A ulvanobiuronic acid 3-sulfate (28%)

SPs: sulfated polysaccharides; SPCr: sulfated polysaccharide of *Caulerpa racemosa*; SPCl: sulfated polysaccharide of *Caulerpa lentillifera*; and nd = not detected. The same letter (a) in the same column indicates a value that is not significantly different (*p* > 0.05) based on the independent-*t* test. * All observed monosaccharides were identified using HPLC-UV-Vis/MS and compared with authentic standards.

**Table 2:** IC_50_ values (μg/mL) exhibited by the SPs for breast, leukemia, colorectal, hepatoma, and normal (primary blood monocular cells [PBMC] and human epithelial) cell lines.

SPs Sample	Colorectal	Hepatoma		Breast Cancer Cell Lines			Leukemia		Control Cell Lines	
	HCT-8	Hep G2	KAIMR C1	MDA-MB-231	MCF-7	KG-1a	K-562	HL-60	Human Epithelial	PBMC
SPCr	1007.50	175.10	320.10	550.60	201.50	403.90	1200.50	1615.50	1260.80	5005.45
SPCl	482.10	1690.00	401.40	310.90	330.53	950.35	1001.70	306.90	3065.10	6095.00
M/ Control	0.201	0.150	0.955	0.890	1.850	0.185	0.458	1.056	0.122	0.189

SPs: sulfated polysaccharides; SPCr: sulfated polysaccharide of *Caulerpa racemosa*; SPCl: sulfated polysaccharide of *Caulerpa lentillifera*; and M/Control: mitoxantrone or positive control. Values highlighted in green indicate potential as an anticancer agent.

## Data Availability

The data sets generated and/or analyzed in this study are available in the manuscript or can be requested from the author (F.N.) upon reasonable request.

## Supplementary Materials

The following supporting information can be downloaded at: https://www.mdpi.com/article/10.3390/molecules28114531/s1. Table S1: The structural visualization of observed methylated sugars in SPs. Table S2: ANOVA Analysis between Biological Activities of SPs and Controls.
