# Supplementary material for: New Insight on In Vitro Biological Activities of Sulfated Polysaccharides from Ulvophyte Green Algae
Source: Molecules. 2023 Jun 2;28(11):4531. doi: 10.3390/molecules28114531 (PMC10254825; doi:10.3390/molecules28114531)
Supplement: Supplementary file 1 [file molecules-28-04531-s001.zip › Supplementary Table S1. The structural visualization of observed methylated sugars in SPs.pdf]

Supplementary File

Table S1: The structural visualization of observed methylated sugars in SPs.

Sample	Observed Methylated Sugars	2D	3D
**SPCr**	2,3-di-*O*-methyl-1,4,5-tri-*O*-acetyl arabinitol; (2R,3S,4R)-3,4-dimethoxypentane-1,2,5-triyl triacetate; **Chemical Formula**: $C_{13}H_{22}O_8$; **Exact Mass**: 306.13; **Molecular Weight**: 306.31 **m/z**: 306.13 (100.0%), 307.13 (14.1%), 308.14 (2.6%); **Elemental Analysis**: C, 50.97; H, 7.24; O, 41.79.	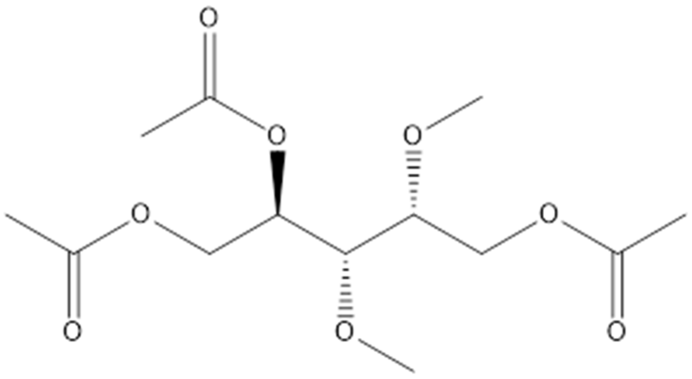	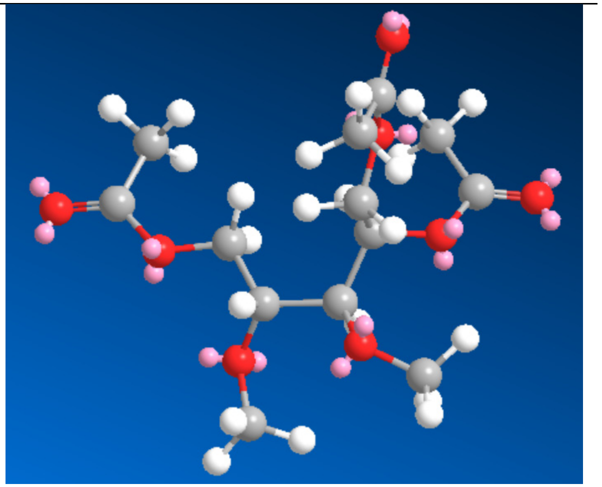
	2,3,4,6-tetra-*O*-methyl-*D*-mannopyranose; (3S,4S,5R,6R)-3,4,5-trimethoxy-6-(methoxymethyl)tetrahydro-2H-pyran-2-ol; **Chemical Formula**: $C_{10}H_{20}O_6$; **Exact Mass**: 236.13; **Molecular Weight**: 236.26; **m/z**: 236.13 (100.0%), 237.13 (11.3%), 238.13 (1.8%); **Elemental Analysis**: C, 50.84; H, 8.53; O, 40.63.	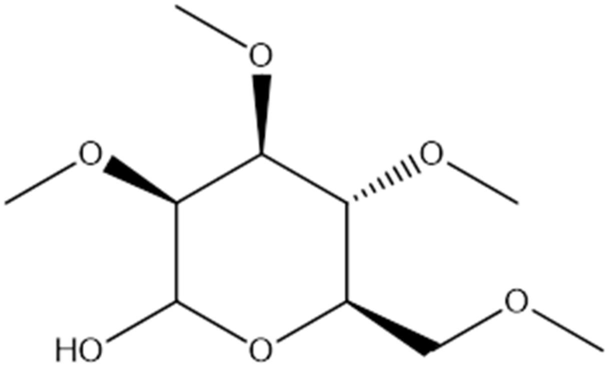	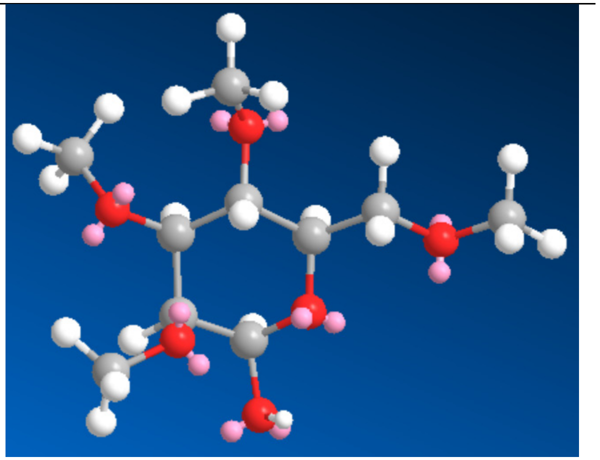

**SPCl**	2,3,4-tri-*O*-methyl-1,5-di-*O*-acetyl xylitol; (2R,3r,4S)-2,3,4-trimethoxypentane-1,5-diyl diacetate; **Chemical Formula**: $C_{12}H_{22}O_7$; **Exact Mass**: 278.14; **Molecular Weight**: 278.30; **m/z**: 278.14 (100.0%), 279.14 (13.5%), 280.14 (2.2%); **Elemental Analysis**: C, 51.79; H, 7.97; O, 40.24.	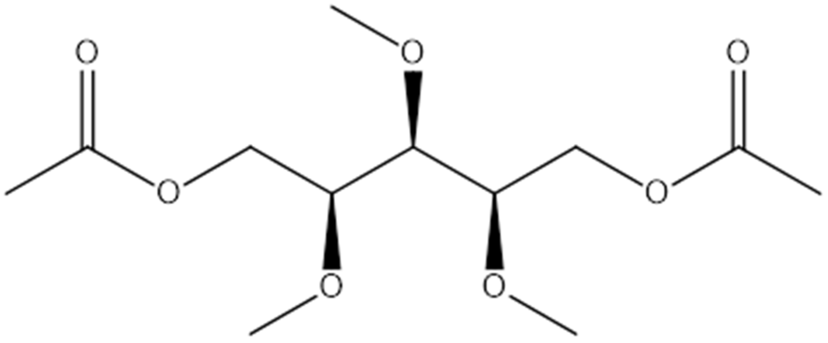	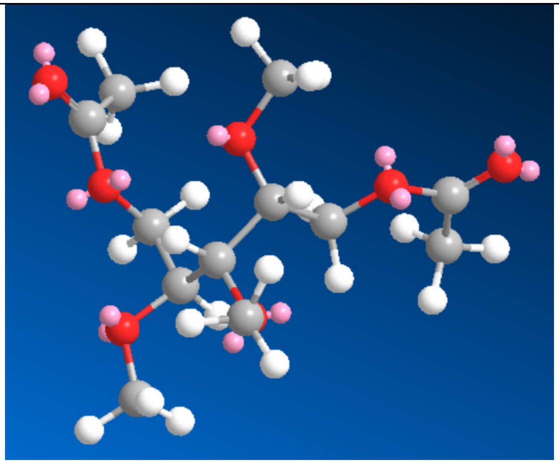
	2,3,4,6-tetra-*O*-methyl-*D*-galactopyranose; (3R,4S,5S,6R)-3,4,5-trimethoxy-6-(methoxymethyl)tetrahydro-2H-pyran-2-ol; **Chemical Formula**: $C_{10}H_{20}O_6$; **Exact Mass**: 236.13; **Molecular Weight**: 236.26; m/z: 236.13 (100.0%), 237.13 (11.3%), 238.13 (1.8%); **Elemental Analysis**: C, 50.84; H, 8.53; O, 40.63.	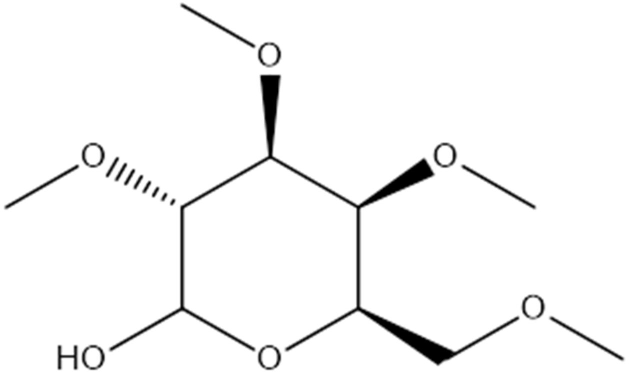	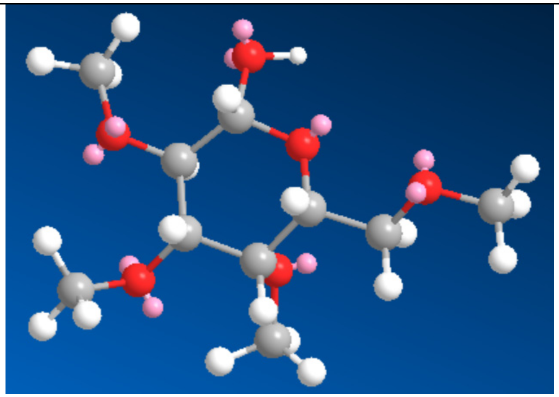
